# Using theatre in education in a traditional lecture oriented medical curriculum

**DOI:** 10.1186/1472-6920-9-73

**Published:** 2009-12-15

**Authors:** Pemra C Ünalan, Arzu Uzuner, Serap Çifçili, Mehmet Akman, Sertaç Hancıoğlu, Hans O Thulesius

**Affiliations:** 1 Dept of Family Medicine, Marmara University Medical Faculty, Tıbbiye Cad. Haydarpasa, 34716 Istanbul, Turkey; 2 General Practitioner, Yukarıçiğil Primary Care Unit, Konya, Turkey; 3 Dept of Clinical Sciences Malmö, Division of Family Medicine, Lund University, Sweden; 4 Welfare Research and Development Centre of Southern Smaland, Box 1223, SE-351 12 Växjö, Sweden

## Abstract

**Background:**

Lectures supported by theatrical performance may enhance learning and be an attractive alternative to traditional lectures. This study describes our experience with using theatre in education for medical students since 2001.

**Methods:**

The volunteer students, coached by experienced students, were given a two-week preparation period to write and prepare different dramatized headache scenarios during three supervised meetings. A theatrical performance was followed by a student presentation about history taking and clinical findings in diagnosing headache. Finally, a group discussion led by students dealt with issues raised in the performance. The evaluation of the theatre in education lecture "A Primary Care Approach to Headache" was based on feedback from students.

**Results:**

More than 90% of 43 responding students fully agreed with the statement "Theatrical performance made it easier to understand the topic". More than 90% disagreed with the statements "Lecture halls were not appropriate for this kind of interaction" and "Students as teachers were not appropriate". Open-ended questions showed that the lesson was thought of as fun, good and useful by most students. The headache questions in the final exam showed results that were similar to average exam results for other questions.

**Conclusion:**

Using theatrical performance in medical education was appreciated by most students and may facilitate learning and enhance empathy and team work communication skills.

## Background

Only our kind of art, soaked as it is in the living experiences of human beings, can artistically reproduce the impalpable shadings and depths of life. Only such art can completely absorb the spectator and make him both understand and also inwardly experience the happenings on the stage, enriching his inner life, and leaving impressions which will not fade with time - Konstantin Stanislavski, "An Actor Prepares" 1936

Traditional lectures often suppress critical thinking and fail to address differences in student learning styles. They rarely put knowledge into practice and students are passive recipients. Art however, stimulates curiosity and creativity [1]. Art can reproduce real life, arouse imagination, and give access to the complexities of illness. As it entertains, it enhances an inwardly understanding that does not easily fade with time and especially humour may enhance memory [2]. Thus, it has been reported [3] that for many people basic health information comes from entertainment television. Theatre has also been used in medical education either in helping students to understand life-threatening situations or medical humanities [1-5]. Lectures supported by theatrical performances may enhance knowledge acquisition and be an attractive alternative to traditional lectures. Applying literature and "theatre" to medical practice and medical school education is not new [4,5]. Apart from that, numerous studies have explored the usefulness of peer education [6]. When teachers allow adequate time for feedback and reflection, engaging students in role playing and peer education, this promotes active learning [7].

We have recognized theatre in medical education as a tool to facilitate teaching not only of communication skills and history taking, but also of common clinical problems. Thus, we have been using this method to teach a clinical approach to headache for third year medical students since 2001. A curriculum reform in Marmara University Medical Faculty 1999-2000 produced mainly traditionally oriented teaching structures and lecture-based forms of instruction. Interactive types of teaching and innovative concepts were judged as more entertaining than effective. So we needed to evaluate the effects of theatre in medical education and peer education in large group sessions. In this study we present experiences of using theatrical performances to teach "A Primary Care Approach to Headache" within a course on nervous system disorders in the third year of medical school.

## Methods

We have applied theatre in medical education lessons to let the audience define and discern different clinical presentations of headache at our medical school since 2001. The dramatized stories are supposed to realistically reflect daily life, but sometimes headache types are presented in an unrealistic way so that each primary headache type was presented as a "finalist" in a "most serious headache contest".

Groups of 4 or 5 volunteer students, supervised by teachers and experienced students from higher classes have been given two weeks to prepare dramatized presentations of different types of headaches using scenarios written by themselves. Usually 3-4 supervised meetings have been sufficient to give guidance in form of rehearsing and coaching. The presentations have been live performances or video recordings and shown to peer students. In 2006 four students volunteered for three supervised preparation meetings. In the first meeting aims, objectives and the lesson format were explained. Video recordings of theatrical presentations from previous years were shown, and a folder of references regarding headache classification, aetiology, clinical presentations and differential diagnosis was provided. In the second meeting the play and the presentation were planned, and dramatic techniques and the script was discussed. In the third meeting the theatrical performance was rehearsed. Every year, depending on the interest of the student group, different scripts were written and different concepts, music or costumes were used. The lecture "A Primary Care Approach to Headache" had two parts each lasting 50 minutes. The students' theatrical performance lasted for approximately 20 minutes and took place in the first part. The audience was asked to take notes about the characteristics of the dramatized headache stories. After the performance, the differences among the "headache stories" were discussed for 20 minutes. In the discussion, run by the students and monitored by teachers, students reflected their objectives on possible histories of the presented illness and developed further questions about the case presented. A 10-minute slide presentation ended the first part of the lesson. In the second part, teachers lectured about the treatment plans and follow-ups of headache patients referring to the dramatized cases.

Ethical approval for the study was not required according to the current regulations at our university. But an approval to conduct the study was obtained from the Educational Board of Coordinators of the Medical Faculty.

### Evaluation

A 1-4 Likert scale questionnaire with, 1 "disagree" to 4 "fully agree" (Table 1), and semi-structured open-ended questions were given to the students in the audience (the observing students) and the performing students separately. The open-ended questions were: "What do you think about the lesson and teaching method?" and "Please give your positive and negative impressions of the lesson". Performing students were asked to write their expectations of the theatre in education lesson, their thoughts about the preparation period, and the effect of the lesson on their learning. The questionnaires were handed out and collected so that the students remained anonymous. The effectiveness of the lesson was assessed by comparing rates of correct answers to multiple-choice questions in the final exam following the course.

**Table 1:** Attitudes of students in the audience (N = 43).

	Students' responses (%)		
	
Statements	disagree	agree	fully agree
"Role-plays made it easier to understand the topic"	2	6	92

"The performing students were successful"	0	7	93

"Using theatre in medical education was useful"	2	9	89

"Using theatre in medical lectures was fun"	2	17	81

"Time for the lesson was sufficient"	2	26	72

"Students as teachers is not appropriate"	95	2	3

"Lecture halls are not appropriate for interaction"	98	2	0

*Row percentages are used.

## Results

Out of 49 students (20 women and 19 men, median age 21, age range 20-23 years), 43 (88%) responded to all of the Likert scale questions (Table 1), and 32 (65%) wrote open-ended responses to the semi-structured questions. More than 90% of the 43 students fully agreed to the statements "Performing students were successful" (93%), and "Role-plays made it easier to understand the topic" (92%). More than 90% disagreed with the statements "Lecture halls are not appropriate for such interactions" (98%) and "Students as teachers were not appropriate" (95%). The statement with the lowest full agreement was "Time for the lesson was sufficient" (72%) followed by "Using theatre in medical education was fun" (81%), and "Using theatre in medical education was useful" (89%).

### Open Ended Questions

#### Observing students' responses

The open-ended responses for 32 students in the audience revealed that the theatre in education lessons generally were thought of as fun, good and useful by 30 students (30/32, 94%); "It was a combination of fun and education", "Method was very good...both funny and meaningful... to see my friends as teachers increased my self confidence", "I saw the patient in his own life not in the clinic", "For the first time in my medical education, I felt myself as I am listening to a patient and as I am a physician". The second most mentioned positive impression was that the lesson provided "sticky", "permanent", and sustainable knowledge (14/32, 44%); "Showing it with gestures and mimicry increases the amount of information that sticks in my mind", "The learning is permanent", "I will remember my friends' cues for the next years. It was interesting to hear such words from them. I think this is a very sustainable knowledge". Two students (2/32, 6%) were critical of the theatre in education lesson: "Why did we spend these hours with plays? It is nonsense", "...What is the outcome of such a lesson for a medical student? I can't understand". Some negative aspects were raised about the second lecturing part, perceived as "boring" by 3/32 students (10%).

#### Performing students' responses

All of the 4 students mentioned the humanitarian perspective, the drive to read more and fun of using theatre in the medical lesson. The other common item was the sustainable knowledge they learned; "We had fun and learned a lot during our group work", "I got a chance to know my friends better as we spend many hours together for this study", "I felt myself closer to the patient than the physician", "It was boring when I saw the document files that the teachers prepared and gave us initially. But I need to read them while writing the scenario", "Such techniques are useful in terms of remembering because of the repetitions and the visual material", "I will not forget the funny story (scenario) we wrote together for the rest of my life...This collaboration was very enjoyable!", "I will remember these stories forever. Because they are my stories!". The time consuming aspect was emphasized by the audience students: "It was more time consuming than we thought".

### Final Exam

The total number of students who took the final exam was 136. As it is not compulsory to attend all classes, 49 students took the evaluated theatrical headache lecture and 4 students were coached and performed the play in the lecture. The teachers of the headache lecture prepared one short essay type question and one multiple choice question which directly related to the educational objectives of the lesson in the final exam. The number of students who answered the questions correctly was 104 (76%) for the essay question and 82 (60%) for the multiple choice question. Of students attending the theatrical lecture, 84% answered the essay question correctly as compared with 72% for non-attendees (p = 0.15, Fisher's exact test). For the MCQ these figures were 66% and 57% (p = 0.47, Fisher's exact test). The discrimination indexes of one MCQ and one essay question relating to the headache lecture were +0.27 and +0.35 respectively while the average discrimination index of the total 90 questions in the final exam was +0.27 (range -0.03 to +0.54).

## Discussion

In this study we report the use of theatrical presentations performed by medical students in a lecture about headache to make medical students more active as learners. More than 90% of participating students agreed that the theatrical performance made it easier to understand the topic. Open-ended questions showed that the lesson was thought of as fun, good and useful by most students who also suggested that this type of lesson promoted "sustainable" or "sticky" knowledge both for performing and observing students. The headache questions in the final exam showed results that were similar to average exam results for other questions. Even though a higher percentage of the students with correct answers to the headache questions had participated in the theatrical headache lecture the difference was not statistically significant.

Some of the performing students in this study were more involved with the patients' stories than with the physicians' roles in the scenarios. Similar understanding of the illness experience and greater empathy for patients were observed by Shapiro et al. They used two one-person shows, dramatically addressing AIDS and ovarian cancer [8,9].

In our study the teachers coached students not only to be good at presentations but also to understand the roles of a teacher. After this group experience, performing students were used to request mentorship from the teachers on other subjects, too. The study thus worked as an icebreaking activity between teachers and students in crowded classes. The performing students need to collaborate for the theatrical presentations promoted mutual respect and cooperation between students and their teachers, and enhanced communication skills within a teamwork setting.

Contrary to the traditional "knowledge transmission view of learning" some authors describe a different perspective focusing on knowledge construction and social exchange [10]. When students are encouraged to set their own goals, and take more responsibility for learning, then teachers become facilitators of learning rather than knowledge providers [11]. Also, shared group experiences of attending a theatrical performance may strengthen bonds among participants and facilitate open communication among performing students [8]. Parallel to these resolutions students in our study were encouraged to set the scenarios and performances in a group work which was accepted to be collaborative and enjoyable in the performing students' own words. As it is emphasized by Lycke et al. group learning processes can promote self-regulated learning and enhance more active engagement in group activities than traditional programs [12].

In some of their qualitative studies Baerheim and Alraek gave the individual student a group-based opportunity to reflect on possible consultation strategies through an actress acting as a patient. Students that were "learning through reflection", "improving a humanistic manner" and "utilizing theatrical tools to facilitate this reflection" were main arguments [13,14]. Some other researchers noted that the support performing students received from other students and from teachers facilitated student centered learning and promoted student confidence [15-17]. Familiarizing the students with the topic, providing resources and information, allowing students to ventilate their feelings, offering counselling to use their own skills, and encouraging were all suggested means to facilitate the students' preparation. The supervising teachers in the present study used all of these interventions. This type of tutorial support may be one of the reasons for the performing students' positive feedback.

## Limitations

The results of our evaluation suggest a fair feasibility of the theatre in education lecture. We cannot claim superiority over traditional lectures since this study had no control group and both performing and observing students were volunteers. Thus, the selection bias was intrinsic. Yet, final exam results of two questions about headache, between the theatre lecture attendees and non-attendees, did at least not suggest a detrimental effect of the theatrical lecture. We also assessed a questionnaire given to lecture participants and found the results to be essentially positive. Incorporating a new educational method in the ordinary didactic program raised some barriers on the students' side. Preparing the presentation meant extra homework and group work hours outside of the program. So the performing students mentioned being stressed both in the preparation and presentation period.

## Conclusions

Integrating theatrical performance created by students and collaborating with them gives the concept of education a different meaning. By using new tools, and looking from the students' viewpoint, we may get ideas of potential new methods that can be applied in the lecture based medical curriculum [16,18]. Medical teachers need creativity and tolerance to new ideas, and different approaches to facilitate learning [19]. Although this intervention was uncontrolled, we suggest that the theatre in medical education used in our "headache lecture" had advantages compared to traditional didactic lectures.

## Competing interests

The authors declare that they have no competing interests.

## Authors' contributions

PCÜ made substantial contributions to the design of the study, data collection, analysis and interpretation of the data as well as drafting the manuscript and revising it critically for intellectual content. AU participated in study design, data collection, analysis and drafting of manuscript. SC and MA made substantial contributions to the design of the study, data interpretation and critical reading. SH conceived the study and collected data. HT helped with data analysis, drafting the manuscript, and revised it critically for intellectual content. All authors read and approved the final manuscript.

## Pre-publication history

The pre-publication history for this paper can be accessed here:

http://www.biomedcentral.com/1472-6920/9/73/prepub
